# Supplementary material for: Associations of Warfarin Use with Risks of Ischemic Cerebrovascular Events and Major Bleeding in Patients with Hyperthyroidism-Related Atrial Fibrillation
Source: Biomedicines. 2022 Oct 22;10(11):2670. doi: 10.3390/biomedicines10112670 (PMC9687236; doi:10.3390/biomedicines10112670)
Supplement: Supplementary file 1 [file biomedicines-10-02670-s001.zip › biomedicines-1905835-supplementary.pdf]

Supplementary Material

Associations of warfarin use with risks of ischemic cerebrovascular events and major bleeding in patients with hyperthyroidism-related atrial fibrillation

Sian-De Liu[1,2,*], Shwu-Jiuan Lin[1,3,*], Chin-Ying Ray[4,5], Fang-Tsyr Lin[6], Weei-Chin Lin[6,7], Li-Hsuan Wang[1,8]

[1] *School of Pharmacy, Taipei Medical University, Taipei, Taiwan*
[2] *Department of Pharmacy, New Taipei Municipal TuCheng Hospital (Built and Operated by Chang Gung Medical Foundation), New Taipei City, Taiwan*
[3] *PhD Program in Clinical Drug Development of Herbal Medicine, College of Pharmacy, Taipei Medical University, Taipei, Taiwan*
[4] *Department of Clinical Pharmacy, Chang Gung Memorial Hospital, Linkou, Taoyuan, Taiwan*
[5] *Heart Failure Center, Chang Gung Memorial Hospital, Linkou, Taoyuan, Taiwan*
[6] *Section of Hematology/Oncology, Department of Medicine, Baylor College of Medicine, Houston, Texas, USA*
[7] *Department of Molecular and Cellular Biology, Baylor College of Medicine, Houston, Texas, USA*
[8] *Department of Pharmacy, Taipei Medical University Hospital, Taipei, Taiwan.*

Corresponding author:
Prof Li-Hsuan Wang, School of Pharmacy, Taipei Medical University, 250 Wu-Hsing St., Taipei 11031, Taiwan. E-mail address: shiuan@tmu.edu.tw

* These authors contributed equally to this work.

Catalog

Table S1 Definitions and codes of comorbidities and outcomes 3
Table S2 Results of univariable and multivariable Robust Cox proportional hazards regression analyses for the outcome of stroke/TIA. 5
Table S3 Results of univariable and multivariable Robust Cox proportional hazards regression analyses for the outcome of major bleeding. 6
Table S4 Sensitivity analysis for new medications after index date. 7
Figure S1 Study flowchart and sensitivity analysis (warfarin versus aspirin users).............8
Table S5 Results of univariable and multivariable Robust Cox proportional hazards regression analyses for the outcome of stroke/TIA among warfarin versus aspirin users ..10

Table S1 Definitions and codes of comorbidities and outcomes

**Diseases**	**ICD-9-CM code**	**ICD-10-CM code**
Atrial fibrillation	427.31	I48.0, I48.1, I48.2, I48.91
Hyperthyroidism	242	E05
Hypothyroidism	243	E03
Ischemic stroke	433, 434, 436, 852, 853	I67.89, I63, I63-I64, G458 G458-459, S01.90XA, S06.4X0AS06.4X0A-S06.4X9A, S06.5X0AS06.5X0A-S06.5X9A, S06.6X0A S06.6X0A-S06.6X9A, S06.340AS06.340A-S06.349A, S06.350AS06.350A-S06.359A, S06.360AS06.360A-S06.369A
Transient ischemic attack	435	G45
Hypertension	401, 402	I10, I11, I12, I13, I14, I15
Myocardial infarction	410, 412	I21, I22, I25.2
Congestive heart failure	398.91, 402.01, 402.11, 402.91, 404.01, 404.03, 404.11, 404.13, 404.91, 404.93, 425.4 425.4-425.9, 428	I09.9, I11.0, I13.0, I13.2, I25.5, I42.0, I42.5I42.5-I42.9, I43, I50, P29.0
Peripheral vascular disease (with atherosclerosis)	093.0, 437.3, 440, 441, 443.1-443.9, 471, 557.1, 557.9	V434, I70, I71, I73.1, I73.8, I73.9, I77.1, I79.0, I79.2, K55.1, K55.8, K55.9, Z95.8, Z95.9
Cerebrovascular disease	362.34, 430-438	G45, G46, H34.0, I60, I61, I62, I63, I64, I65, I66, I67, I68, I69
Diabetes mellitus	250	E11, E10
Chronic kidney disease	580-589	I12, I13, N00-N05, N07, N11, N14, N17-N19, Q61
Chronic obstructive pulmonary disease	490, 491.0, 491.1, 491.2, 491.20-491.22, 491.8, 491.9, 492.0, 492.8, 494, 494.0, 494.1, 496	J40, J41.0, J41.1, J41.8, J42, J43.0-J43.2, J43.8, J43.9, J44.0, J44.1, J44.9, J47.0, J47.1, J47.9
Any malignancy, including lymphoma and leukemia, except malignant neoplasm of skin	140-172, 174-195.8, 200-208, 238.6	C00-C26, C30-C34, C37-C41, C43, C45-C58, C60-C76, C81-C85, C88, C90-C97
Rheumatic disease	446.5, 710.0, 710.1, 710.2, 710.3, 710.4, 714.0, 714.1, 714.2,714.8, 725	M05, M06, M315, M32, M33, M34, M351, M353, M36.0

Hyperlipidemia	272.0, 272.1, 272.2, 272.3, 272.4, 272.9	E780, E781, E782, E783, E784, E756, E785, E78.7
Cardiomyopathy (dilated, hypertrophic, restrictive, obstructive)	425	I42
Thromboembolism (Pulmonary embolism+DVT)	415.19, I26.99 , I26.09, 453.8, 453.9, 453.40, 453.41, 453.42	I82.2, I82.4-I82.9, I82.A, I82.B, I82.C
Valve anomalies	746	Q22, Q23, Q24
**Major bleeding**		
Intracranial	430, 431, 432.0, 432.1, 432.9, 852.0, 852.2, 852.4, 853.0	I60, I61, I62, S06.340A S06.340A-S06.349A, S06.350AS06.350A-S06.359A, S06.360AS06.360A-06.369A, S06.4X0AS06.4X0A-S06.4X9A, S06.5X0AS06.5X0A-S06.5X9A, S06.6X0AS06.6X0A-S06.6X9A
Gastrointestinal	530.7, 531, 531.2, 531.4, 531.6, 532, 532.2, 532.4, 532.6, 533, 533.2, 533.4, 533.6, 534, 534.2, 534.4, 534.6, 569.3, 535.01, 535.11, 535.21, 535.31, 535.41, 535.51, 535.61, 535.71, 537.83, 537.84, 562.02, 562.03, 562.12, 562.13, 569.85, 578	K22.6, K25-K28 , K29.01, K29.21, K29.31, K29.41, K29.51, K29.61, K29.71, K29.81, K29.91, K31.811, K31.82, K52.81, K55.21, K56.60, K57.01, K57.11, K57.13, K57.21, K57.31, K57.33, K57.81, K57.91, K57.93, K62.5, K92.0-K92.2
Other sites	336.1, 363.6, 372.72, 376.32, 377.42, 379.23, 593.81, 866.01, 866.02, 866.11, 866.12, 719.1, 729.92, 423.0, 772.5	G95.11, G95.19, H05.23, H11.3, H31.3, H43.1, H47.02, I31.2, M25.0, N28.0, P54.4, S31.001A, S37.011A, S37.012A, S37.019A, S37.021A, S37.022A, S37.029A, S37.031A, S37.032A, S37.039A, S37.041A, S37.042A, S37.049A, S37.051A, S37.052A, S37.059A

DVT deep vein thrombosis, *ICD-9-CM* International Classification of Disease-Ninth Revision- Clinical Modification, *ICD-10-CM* International Classification of Disease-Tenth Revision-Clinical Modification

Table S2 Results of univariable and multivariable Robust Cox proportional hazards regression analyses for the outcome of stroke/TIA

	**crude HR (95% CI)**	**p value**	**adjusted HR (95% CI)**	**p value**
Age, years	1.04 (1.02–1.06)	<.0001	1.07 (1.02–1.12)	0.010
Gender/Male	0.60 (0.34–1.07)	0.082	0.74 (0.30–1.78)	0.494
CHA2DS2-VASc	1.62 (1.31–2.01)	<.0001	0.56 (0.26–1.22)	0.147
Hypertension	3.85 (1.99–7.44)	<.0001	2.86 (0.88–9.35)	0.082
Congestive heart failure	2.69 (1.47–4.91)	0.001	4.30 (1.15–16.09)	0.030
Peripheral vascular disease	4.98 (1.55–15.94)	0.007	6.01 (1.56–23.11)	0.009
Cerebrovascular disease	1.27 (0.19–8.30)	0.807	NA	NA
Diabetes mellitus	2.49 (1.28–4.83)	0.007	2.23 (0.64–7.79)	0.208
Chronic kidney disease	3.53 (0.78–16.05)	0.103	NA	NA
Chronic obstructive pulmonary disease	1.71 (0.83–3.53)	0.149	NA	NA
Hyperlipidemia	1.23 (0.52–2.88)	0.639	NA	NA
Amiodarone	1.08 (0.50–2.37)	0.842	NA	NA
Aspirin	4.55 (2.27–9.13)	<.0001	3.42 (1.37–8.54)	0.008
Clopidogrel	1.35 (0.17–10.55)	0.776	NA	NA
NSAID	2.09 (0.86–5.08)	0.103	NA	NA
Statin	1.42 (0.53–3.79)	0.482	NA	NA

CI confidence interval, *HR* hazard ratio, *NA* not available, *NSAID*, non-steroid anti-inflammatory drug, *TIA* transient ischemic attack

Table S3 Results of univariable and multivariable Robust Cox proportional hazards regression analyses for the outcome of major bleeding

	crude HR (95% CI)	p value	adjusted HR (95% CI)	p value
Age, years	1.02 (1.00–1.03)	0.020	1.00 (0.97–1.03)	0.950
Gender/Male	0.79 (0.53–1.18)	0.247	1.25 (0.67–2.34)	0.485
CHA2DS2-VASc	1.32 (1.17–1.49)	<.0001	1.24 (0.74–2.10)	0.413
Hypertension	2.93 (2.02–4.23)	<.0001	2.11 (1.15–3.88)	0.016
Congestive heart failure	1.53 (1.01–2.31)	0.044	0.99 (0.35–2.81)	0.987
Peripheral vascular disease	2.00 (0.87–4.61)	0.103	NA	NA
Cerebrovascular disease	0.88 (0.27–2.84)	0.832	NA	NA
Diabetes mellitus	2.12 (1.37–3.26)	0.001	1.19 (0.48–2.96)	0.706
Chronic kidney disease	1.79 (0.73–4.38)	0.200	NA	NA
Chronic obstructive pulmonary disease	2.20 (1.40–3.47)	0.001	0.85 (0.50–1.45)	0.552
Hyperlipidemia	2.42 (1.39–4.21)	0.002	1.30 (0.73–2.31)	0.377
Cardiomyopathy	0.57 (0.18–1.84)	0.350	NA	NA
Amiodarone	0.93 (0.59–1.45)	0.732	NA	NA
Aspirin	2.65 (1.87–3.74)	<.0001	1.85 (1.20–2.85)	0.006
Clopidogrel	2.76 (0.77–9.77)	0.117	NA	NA
NSAID	1.55 (0.91–2.65)	0.111	NA	NA
Statin	2.23 (1.03–4.85)	0.043	0.77 (0.35–1.71)	0.527

CI confidence interval, *HR* hazard ratio, *NA* not available, *NSAID*, non-steroid anti-inflammatory drug

Table S4 Sensitivity analysis for new medications after index date

	Warfarin users N=90	Warfarin nonusers N=168	*P* value
**Number of new users**			
received DOAC (n, %)	19 (21.1%)	12 (7.1%)	0.001
received antiplatelet medication (n, %)	6 (6.7%)	10 (6.0%)	0.821
**Outcomes after excluding new medication**			
aHR for Stoke/TIA (95% CI)	1.18 (0.53–2.64)	Reference	0.693
aHR for Major bleeding (95% CI)	0.95 (0.58–1.55)	Reference	0.822

aHR adjusted hazard ratio, *CI* confidence interval, *DOAC* direct oral anticoagulants, *TIA* transient ischemic attack

Figure S1 Study flowchart and sensitivity analysis (warfarin versus aspirin users)

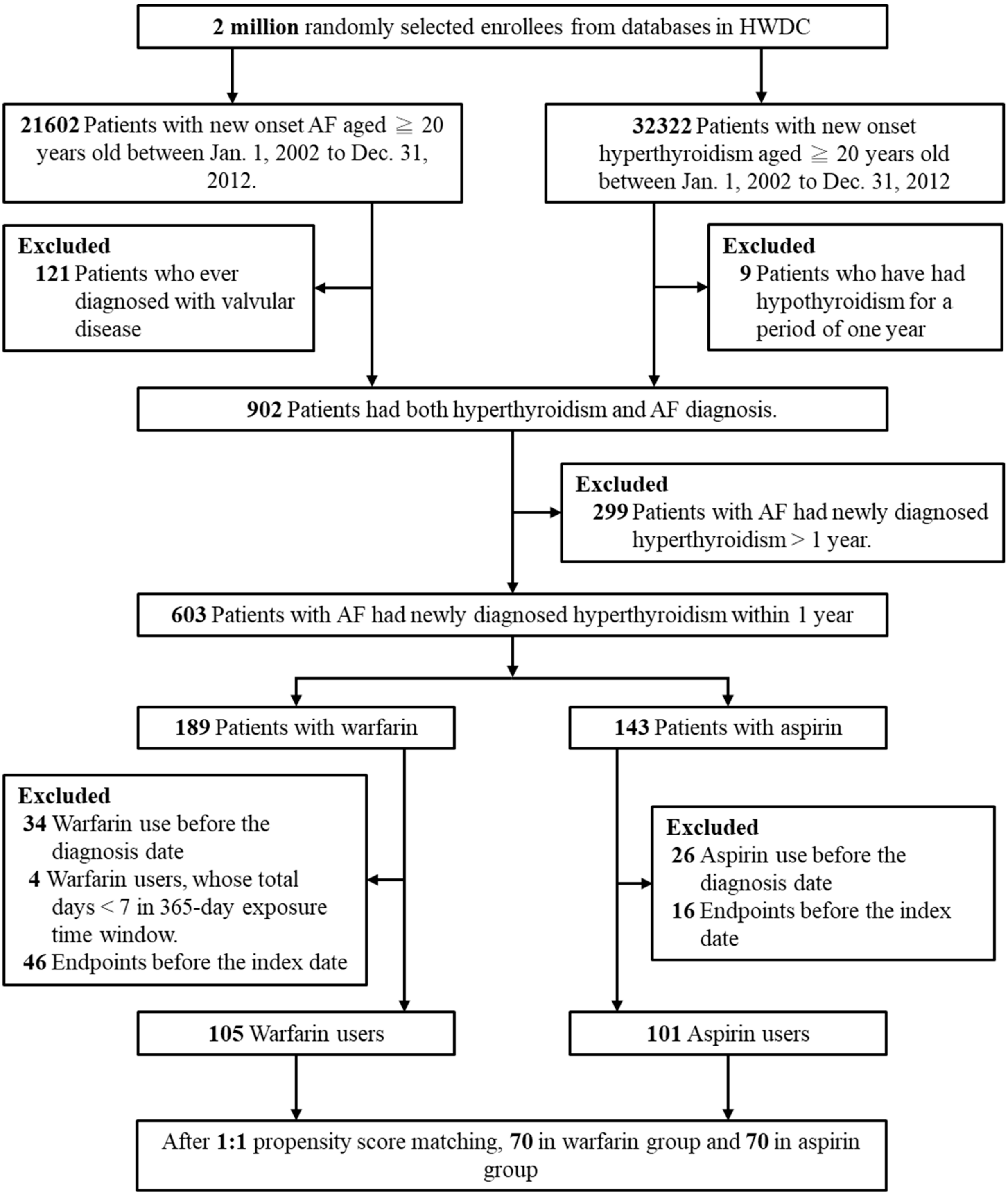

AF atrial fibrillation, *HWDC* Health and Welfare Data Science Center

Table S5 Results of univariable and multivariable Robust Cox proportional hazards regression analyses for the outcome of stroke/TIA among warfarin versus aspirin users

	crude HR (95% CI)	p value	adjusted HR (95% CI)	p value
Age, years	1.04 (1.01–1.07)	0.007	1.09 (0.99–1.21)	0.090
Gender/Male	0.87 (0.41–1.86)	0.723	0.57 (0.08–3.95)	0.571
CHA2DS2-VASc	1.57 (1.22–2.02)	0.0004	0.32 (0.07–1.47)	0.144
Hypertension	5.04 (2.27–11.18)	<0.0001	9.52 (1.18–76.58)	0.034
Congestive heart failure	2.24 (1.12–4.51)	0.023	8.46 (0.86–83.41)	0.067
Peripheral vascular disease	2.50 (0.23–27.84)	0.455	NA	NA
Diabetes mellitus	2.94 (1.46–5.93)	0.003	2.89 (0.45–18.58)	0.265
Chronic kidney disease	1.26 (0.15–10.73)	0.833	0.88 (0.05–15.58)	0.929
Chronic obstructive pulmonary disease	1.78 (0.64–4.95)	0.272	NA	NA
Hyperlipidemia	0.89 (0.20–3.91)	0.874	NA	NA
Amiodarone	0.95 (0.35–2.59)	0.925	NA	NA
Clopidogrel	12.82 (7.11–23.10)	<0.0001	7.16 (1.04–49.35)	0.046
NSAID	1.62 (0.60–4.36)	0.338	NA	NA
Statin	4.49 (1.07–18.82)	0.040	2.16 (0.33–14.21)	0.424

CI confidence interval, *HR* hazard ratio, *NA* not available, *NSAID*, non-steroid anti-inflammatory drug, *TIA* transient ischemic attack
